# The clinical value of pediatric laparoscopic surgery using umbilical S-shaped incision

**DOI:** 10.3389/fped.2026.1902155

**Published:** 2026-07-09

**Authors:** Shuaishuai Han, Chunyan Wang, Qingshuang Wu, Shuzhen Lin, Jizhou Shi

**Affiliations:** 1 Department of Pediatric Surgery, Shengli Oilfield Central Hospital, Dongying, China; 2 Department of Pediatric Surgery, Dongying District People’s Hospital, Dongying, China

**Keywords:** children, cosmetic outcome, minimally invasive surgery, pediatric laparoscopic surgery, transumbilical approach, umbilical S-shaped incision

## Abstract

**Objective:**

To explore the clinical efficacy, safety and application value of umbilical S-shaped incision in pediatric laparoscopic surgery.

**Methods:**

A retrospective analysis was conducted on the clinical data of children who underwent surgery via umbilical S-shaped incision in the Department of Pediatric Surgery, Shengli Oilfield Central Hospital from January 2019 to April 2026. The collected data included age, gender, surgical procedures, incision morphology and postoperative complications.

**Results:**

A total of 36 children received umbilical S-shaped incision. The mean age was 6.5 ± 4.4 years old. The surgical procedures comprised 5 cases of laparoscopic choledochal cyst resection combined with hepaticojejunostomy, 5 cases of laparoscopic single-port transumbilical appendectomy, 5 cases of laparoscopic single-site multi-port transumbilical appendectomy, 8 cases of laparoscopic exploration converted to open surgery for small intestinal diverticulectomy, 2 cases of laparoscopic exploration converted to open surgery for enterotomy and foreign body removal, 3 cases of laparoscopic exploration converted to open surgery for small intestinal duplication malformation repair, 2 cases of laparoscopic single-site multi-port transumbilical pyeloplasty, 1 case of laparoscopic retroperitoneal teratoma resection, 3 cases of laparoscopic exploration converted to open surgery for small intestinal lymphangioma resection, 1 case of laparoscopic exploration converted to open surgery for intussusception reduction and small intestinal tumor resection, and 1 case of laparoscopic ovarian teratoma enucleation. All operations were completed successfully. No incision-related complications occurred, and the transumbilical incisions achieved nearly scarless outcomes.

**Conclusion:**

Confined to the umbilicus, the umbilical S-shaped incision allows extracorporeal resection and anastomosis of the small intestine, as well as specimen removal of ovarian teratoma. It does not increase the difficulty of laparoscopic operation, while ensuring concealed incision and satisfactory cosmetic results.

## Introduction

Laparoscopic surgery has become a routine therapeutic approach for a wide range of pediatric surgical diseases involving the gastrointestinal tract, hepatobiliary system, urinary system and tumors (1). Transumbilical laparoscopic single-port surgery (2) and transumbilical laparoscopic single-site surgery (3) represent important developmental trends in pediatric minimally invasive surgery. The core concept is to perform operations via the natural anatomical orifice of the umbilicus to achieve an essentially scarless cosmetic outcome.

Nevertheless, as all surgical instruments are introduced through a single access port, the operative triangulation is lost. The parallel arrangement of instruments, also known as the “chopstick effect”, substantially increases the technical difficulty of surgery. For laparoscopic resection of choledochal cysts in children, digestive tract reconstruction is generally performed extracorporeally, which often requires extending the umbilical incision to approximately 2 cm beyond the umbilical margin. In some cases, the umbilical incision has to be further enlarged for complete retrieval of pathological specimens, leaving noticeable postoperative scars and compromising cosmetic results.

This article introduces an S-shaped umbilical incision technique. Although the superficial skin incision is confined entirely within the umbilicus, a working incision of 4–6 cm in length can be obtained after skin retraction. After placement of a wound protector, the incision is transformed into a circular access with a diameter of around 3–4 cm. Through this access, organs such as the intestines and ovaries can be exteriorized for extracorporeal manipulation, and tissue specimens can also be retrieved smoothly.

## Method

A retrospective analysis was performed on the clinical data of pediatric patients who underwent surgery via umbilical S-shaped incision in the Department of Pediatric Surgery, Shengli Oilfield Central Hospital from January 2019 to April 2026. The collected data included age, gender, surgical procedures, incision morphology and complications (Table 1).

**Table 1:** Baseline characteristics of 36 patients.

No.	Sex	Age	Diagnosis
1	Male	12 years and 11 months	Acute suppurative appendicitis
2	Female	3 years and 11 months	Acute suppurative appendicitis
3	Female	11 years and 1 month	Acute suppurative appendicitis
4	Female	6 years and 2 months	Acute suppurative appendicitis
5	Male	14 years and 2 months	Acute suppurative appendicitis
6	Male	5 years and 3 months	Acute suppurative appendicitis
7	Male	8 years and 6 months	Acute suppurative appendicitis
8	Male	3 years and 2 months	Acute suppurative appendicitis
9	Female	4 years and 10 months	Acute suppurative appendicitis
10	Male	14 years and 11 months	Acute suppurative appendicitis
11	Female	9 years and 8 months	Small intestinal diverticulum
12	Male	7 years and 1 month	Small intestinal diverticulum
13	Male	13 years and 4 months	Small intestinal diverticulum
14	Male	2 years and 9 months	Small intestinal diverticulum
15	Male	10 years and 2 months	Small intestinal diverticulum
16	Male	12 years and 9 months	Small intestinal diverticulum
17	Female	10 years and 3 months	Small intestinal diverticulum
18	Female	8 years and 4 months	Small intestinal diverticulum
19	Female	2 years and 4 months	Choledochal cyst
20	Female	8 months and 21 days	Choledochal cyst
21	Male	7 months	Choledochal cyst
22	Female	2 years and 5 months	Choledochal cyst
23	Female	1 year and 10 months	Choledochal cyst
24	Male	6 years and 7 months	Intestinal duplication malformation
25	Male	6 months	Intestinal duplication malformation
26	Male	4 years and 8 months	Intestinal duplication malformation
27	Male	5 years and 11 months	Hydronephrosis (left side)
28	Female	5 years	Hydronephrosis (left side)
29	Female	7 years and 8 months	Retroperitoneal teratoma
30	Male	5 years and 4 months	Small intestinal mesenteric lymphangioma
31	Male	4 years	Small intestinal mesenteric lymphangioma
32	Female	2 years and 11 months	Small intestinal mesenteric lymphangioma
33	Female	13 years and 10 months	Ovarian teratoma
34	Female	5 months and 24 days	Intussusception; Small intestinal tumor
35	Female	10 months and 27 days	Small intestinal foreign body (water beads)
36	Female	10 years and 4 months	Small intestinal foreign body (Buckyballs)

## Transumbilical single-site appendectomy

**Figure 1:**
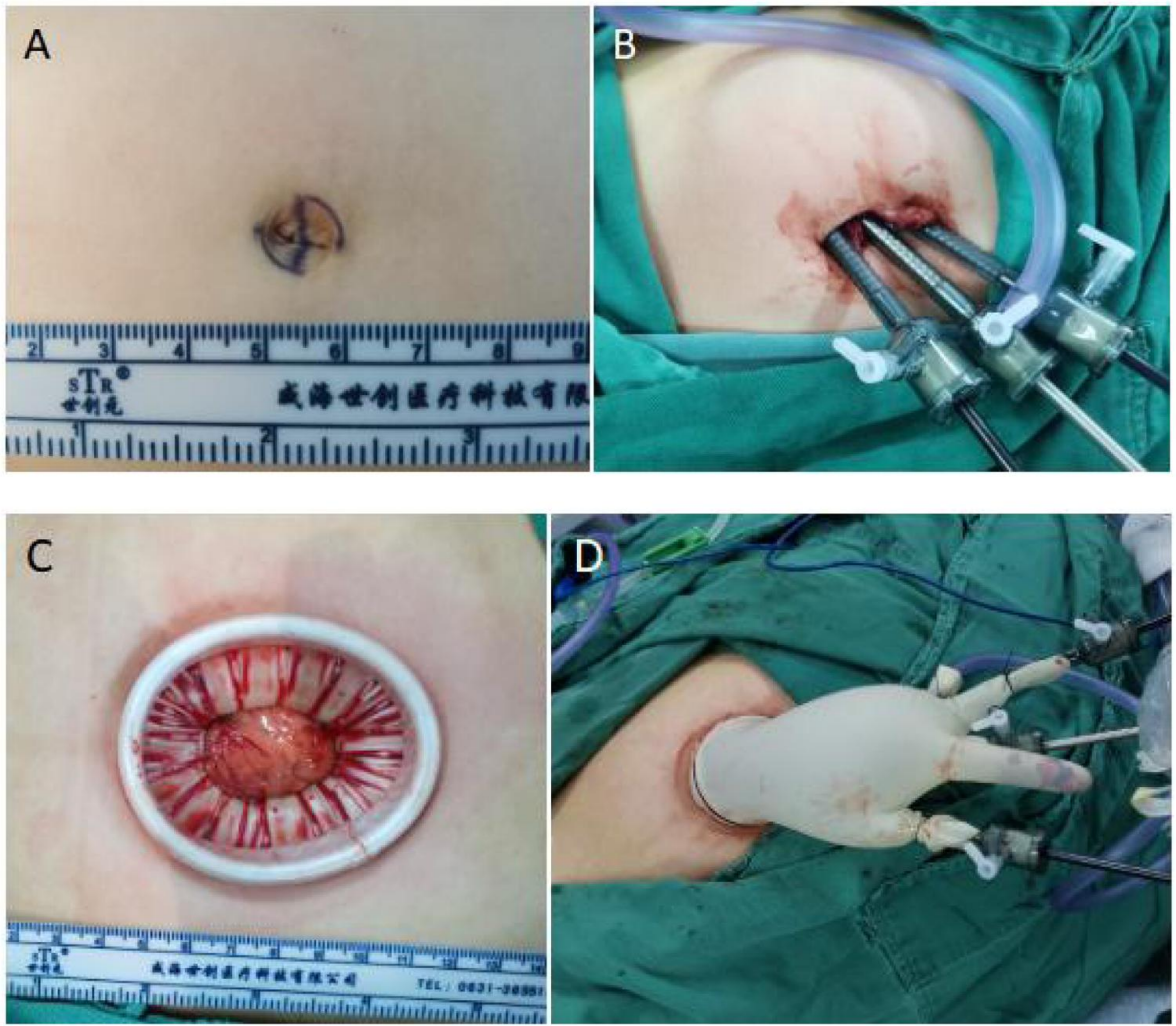
**(A)** Construction of the S-shaped umbilical incision. **(B)** Layout of three trocars for transumbilical single-site multi-port appendectomy after longitudinal stretching of incision. **(C)** Longitudinal cut of umbilical annulus and linea alba to build a 3 cm round operative channel with incision protector. **(D)** Implementation of transumbilical single-port appendectomy assisted by sterile surgical glove.

General endotracheal anesthesia was induced with the patient in the supine position. A transverse incision was made at the mid-umbilicus, and both ends of the incision were extended upward and downward along the umbilical margin for one quarter of the umbilical circumference to form an umbilical S-shaped incision (Figure 1A). After cutting through the skin and subcutaneous tissue, longitudinal traction on the S-shaped incision creates a longitudinal surgical wound. This approach allows the performance of transumbilical single-site multi-port appendectomy (Figure 1B). The spacing between the three trocars is larger than that in conventional transumbilical single-site surgery, which effectively avoids the “chopstick effect”. If the umbilical ring and abdominal linea alba are incised longitudinally and an incision protector is placed, a circular access channel with a diameter of 3 cm is established (Figure 1C). With the assistance of a surgical glove, transumbilical single-port appendectomy can be performed (Figure 1D).

## Laparoscopic exploration converted to open surgery for small intestinal diverticulectomy

**Figure 2:**
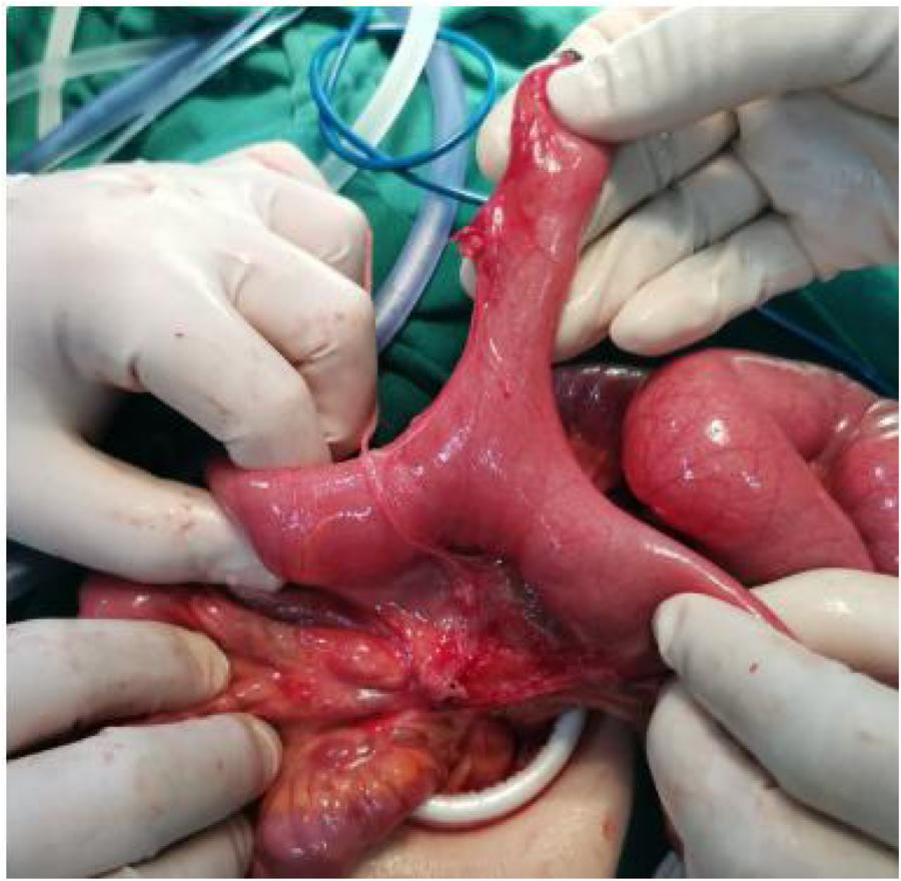
Exteriorization of small intestinal diverticulum via the S-shaped umbilical access for diverticulectomy and intestinal end-to-end anastomosis.

The children presented with painless active hematochezia preoperatively. No bleeding lesions were detected by gastroscopy and colonoscopy. Diverticula were not identified on preoperative ultrasonography and CT scans. General endotracheal anesthesia was administered with the patient in the supine position. An umbilical S-shaped incision was created, followed by longitudinal incision of the umbilical ring and linea alba. An incision protector was inserted, and laparoscopic exploration was performed with the assistance of a surgical glove, which revealed small intestinal diverticula. The diverticula were exteriorized through the incision for diverticulectomy and end-to-end anastomosis of the small intestine (Figure 2).

## Laparoscopic exploration converted to open surgery for intussusception reduction and small intestinal tumor resection

**Figure 3:**
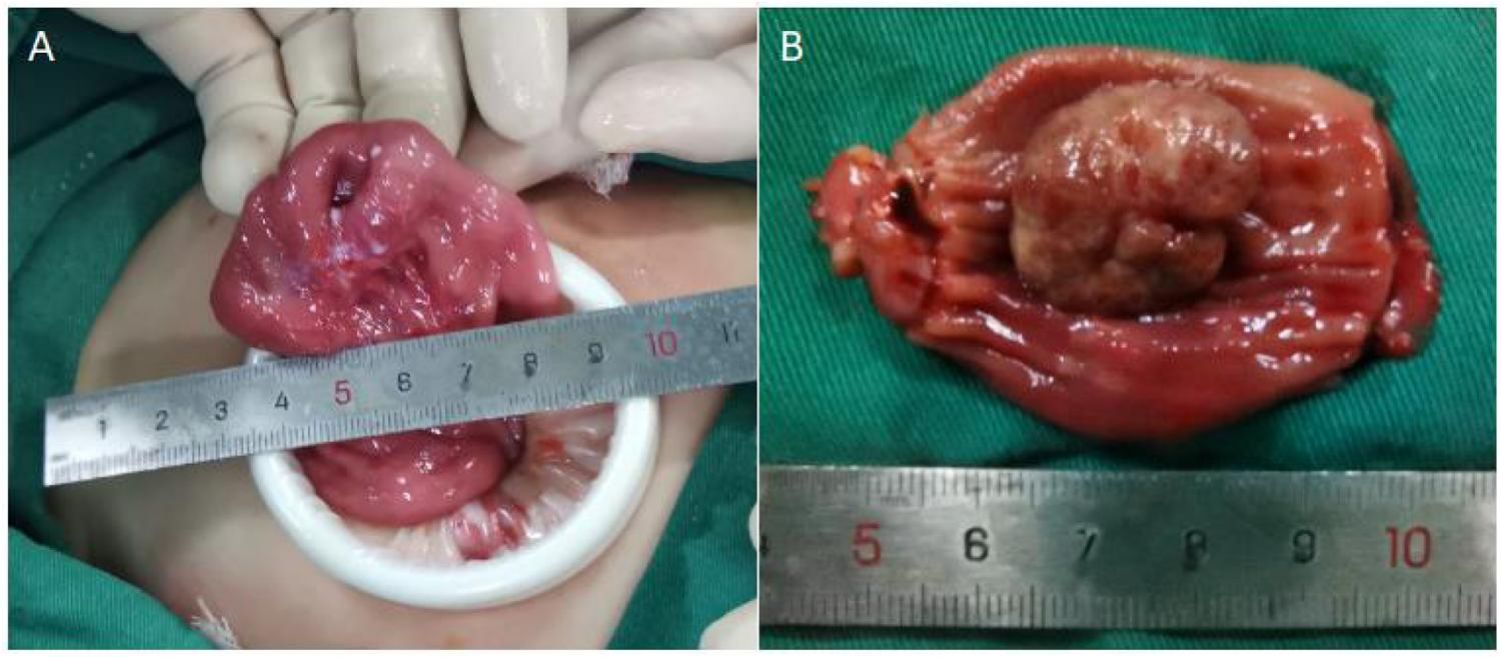
**(A)** Exteriorization of the intussuscepted intestinal segment through the S-shaped umbilical incision. **(B)** Intraoperative identification of small intestinal mass after successful intussusception reduction.

The child was diagnosed with intussusception preoperatively. Reduction with low-pressure air enema failed, and laparoscopic exploration was then scheduled. With the patient placed in the supine position, general endotracheal anesthesia was induced. An umbilical S-shaped incision was made, followed by longitudinal incision of the umbilical ring and linea alba. An incision protector was inserted, and laparoscopic exploration was conducted with the assistance of a surgical glove. Attempts at laparoscopic reduction of intussusception were unsuccessful. The intussuscepted bowel was exteriorized through the umbilical incision for manual reduction, which was accomplished successfully. A small intestinal mass was identified thereafter (Figures 3A,B). The affected intestinal segment was exteriorized via the incision for tumor resection and end-to-end anastomosis of the small intestine.

## Laparoscopic exploration converted to open surgery for resection of small intestinal lymphangioma

**Figure 4:**
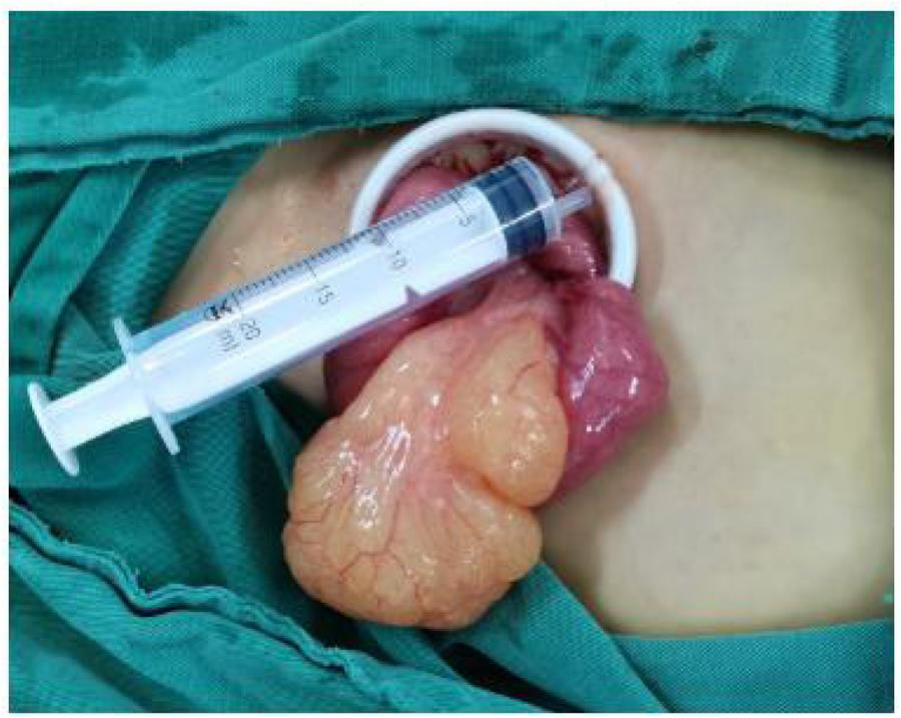
Exteriorization of diseased intestinal tract via the S-shaped umbilical incision for resection of small intestinal lymphangioma and intestinal end-to-end anastomosis.

The child was preoperatively diagnosed with small intestinal lymphatic malformation. General endotracheal anesthesia was induced with the patient in the supine position. An umbilical S-shaped incision was made, followed by longitudinal incision of the umbilical ring and linea alba. An incision protector was placed, and laparoscopic exploration was performed with the assistance of a surgical glove, which confirmed the presence of small intestinal lymphangioma. The affected intestinal segment was exteriorized through the umbilical incision for partial small intestinal resection and end-to-end anastomosis (Figure 4).

## Laparoscopic choledochal cyst resection with hepaticojejunostomy

**Figure 5:**
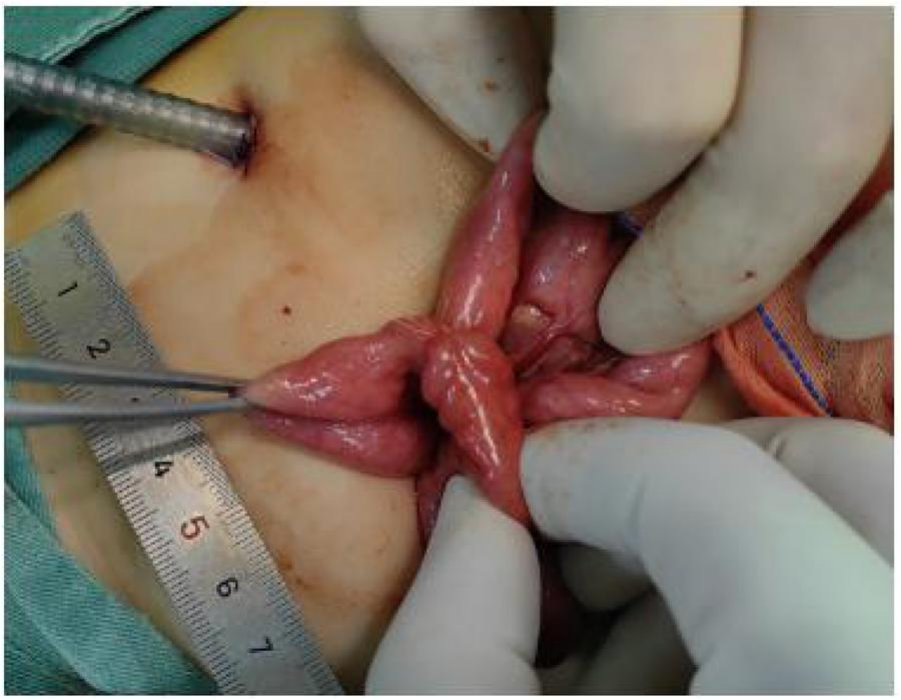
Extraction of jejunum through the S-shaped umbilical incision for intestinal transection and end-to-side anastomosis after choledochal cyst resection.

General endotracheal anesthesia was induced with the patient in the supine position. Trocars were inserted at multiple sites in accordance with standard protocols, including one trocar placed through the umbilical S-shaped incision. After resection of the choledochal cyst, the linea alba beneath the umbilical S-shaped incision was incised longitudinally. The jejunum was exteriorized through the incision for jejunal division and end-to-side anastomosis to reestablish intestinal continuity (Figure 5). Finally, another trocar was reinserted via the umbilical incision to perform laparoscopic hepaticojejunostomy.

## Others

Two cases underwent laparoscopic exploration converted to open surgery for enterotomy and foreign body removal. The foreign bodies were magnetic balls (Buckyballs) and water beads respectively. The surgical procedure was similar to that of laparoscopic exploration converted to open surgery for small intestinal diverticulectomy. The operative steps of laparoscopic exploration converted to open surgery for repair of small intestinal duplication malformation were analogous to the above procedure. The establishment of operative access for transumbilical single-site multi-port laparoscopic pyeloplasty was similar to that of transumbilical single-site multi-port appendectomy. For laparoscopic resection of retroperitoneal teratoma and laparoscopic ovarian teratoma enucleation, the S-shaped incision was mainly used for specimen extraction.

## Results

**Figure 6:**
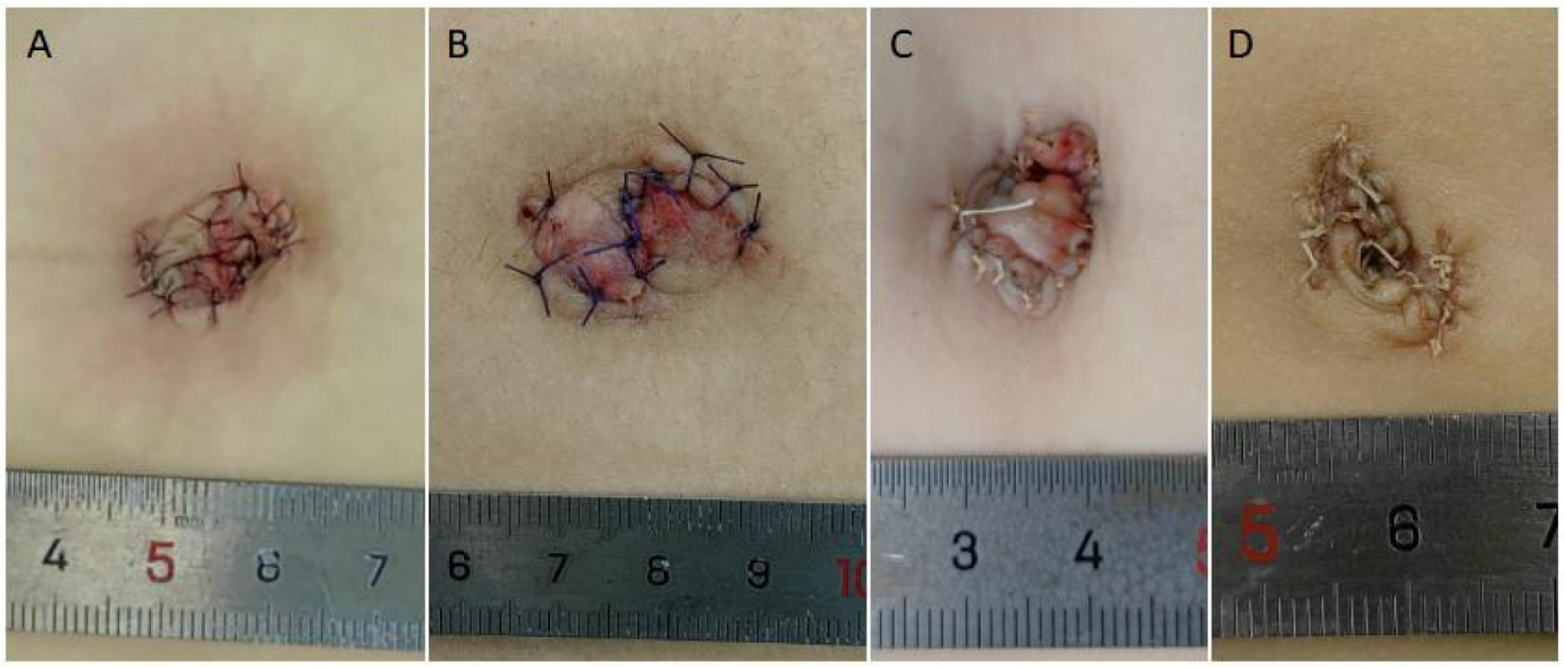
**(A–D)** Representative postoperative cosmetic results of different cases after the S-shaped umbilical incision surgery.

All patients underwent successful surgical procedures with incisions confined to the umbilical region at the end of surgery, achieving excellent cosmetic outcomes with concealed scars (Figures 6A–D). Based on our cumulative clinical experience in umbilical S-shaped incision, the total time required for incision establishment and closure is approximately 20 min, while the total time for incision establishment and closure of the traditional incision is about 10 min. No complications such as incisional hernia or surgical site infection were observed. The S-shaped incision conformed to the natural periumbilical skin folds, allowing for even distribution of tension and effectively reducing the risk of skin edge ischemia and poor wound healing. The temporarily enlarged incision created by intraoperative traction met the requirements for intact specimen extraction and extracorporeal anastomosis while avoiding the trauma associated with conventional longitudinal incisions.

## Discussion

The umbilicus is a natural physiological scar of the human body. In the era of laparoscopic surgery, numerous researchers have explored ways to fully utilize the umbilicus for surgical manipulation, operative field exposure and specimen extraction. Akio Odaka described the use of the transumbilical sliding-window method for omental cyst resection and splenectomy in two pediatric patients, which successfully avoided large surgical incisions (4). Casciola and colleagues reported employing a transverse periumbilical incision for the removal of large specimens (5). Similarly, Yu et al. presented a modified transverse periumbilical incision combined with a homemade transumbilical port to perform laparoendoscopic single-site nephrectomy (6). Nevertheless, the incisions adopted in the above studies all extended beyond the umbilical margin, leaving postoperative scars that could not be confined within the umbilicus. In single-site umbilical laparoscopic appendectomy, the observation port is placed at the midpoint of the umbilicus and two working ports at the umbilical margin, with all three trocars positioned entirely within the umbilical area (7). An identical trocar arrangement has been applied in laparoscopic single-incision triangulated umbilical surgery pyeloplasty (8). However, the extremely short distance between the three trocars inevitably causes the “chopstick effect” and increases the difficulty of surgical manipulation. By contrast, when performing single-site umbilical laparoscopic appendectomy via our designed S-shaped incision, the two working ports can penetrate the muscular layer 1 cm outside the umbilical margin, which partially alleviates the chopstick effect.

Hachisuka proposed transumbilical laparoscopic surgery using an umbilical zigzag skin incision. Shinichi Umeda reported the application of umbilical zigzag incision for combined surgical procedures. Kato et al. (9) and Kaneko et al. (10) adopted the umbilical zigzag incision in adult renal carcinoma surgery, and Yamana et al. used this incision for adult Meckel's diverticulitis surgery (11). Although the zigzag skin incision extends beyond the umbilical ring and provides a larger access channel, it compromises the cosmetic concealment of the incision (12). Amano et al. introduced the umbilical benz incision for reduced-port surgery in children (13). While the benz incision is well concealed within the umbilicus, it offers a shorter operative exposure compared with our S-shaped incision. Another advantage of our S-shaped incision is its favorable compatibility with laparoscopic surgery for pediatric inguinal hernia and hydrocele. Currently, an increasing number of surgeons perform transumbilical single-port and two-port laparoscopy for the treatment of pediatric inguinal hernia (14). If concomitant lesions such as small intestinal diverticula are identified intraoperatively, the existing transverse umbilical incision can be extended and connected to form an S-shaped incision, facilitating *in-vivo* manipulation, extracorporeal specimen resection and intestinal anastomosis. In cases of oversized specimens, the S-shaped incision can be lengthened longitudinally to achieve adequate surgical exposure. Longitudinal incision extension yields better cosmetic outcomes than transverse extension (15).

In addition, this study lacks a prospective control group and fails to conduct a horizontal comparative analysis with traditional umbilical transverse incision, Z-shaped incision and other common surgical methods. Meanwhile, restricted by the retrospective study design, the long-term postoperative follow-up data of patients is insufficient, and the long-term efficacy and long-term cosmetic prognosis of the improved incision cannot be fully verified.

## Conclusion

The umbilical S-shaped incision, while confined to the umbilical region, enables extracorporeal exploration, resection, and reconstruction of small intestine and other organs, as well as specimen extraction. It ensures incision concealment with satisfactory aesthetic outcomes.

## Data Availability

The original contributions presented in the study are included in the article/Supplementary Material, further inquiries can be directed to the corresponding author.
